# Spontaneous and Divergent Hexaploid Triticales Derived from Common Wheat × Rye by Complete Elimination of D-Genome Chromosomes

**DOI:** 10.1371/journal.pone.0120421

**Published:** 2015-03-17

**Authors:** Hao Li, Xiaoxue Guo, Changyou Wang, Wanquan Ji

**Affiliations:** State Key Laboratory of Crop Stress Biology for Arid Areas and College of Agronomy, Northwest A & F University, Yangling, Shaanxi, China; Leibniz-Institute of Plant Genetics and Crop Plant Research (IPK), GERMANY

## Abstract

**Background:**

Hexaploid triticale could be either synthesized by crossing tetraploid wheat with rye, or developed by crossing hexaploid wheat with a hexaploid triticale or an octoploid triticale.

**Methodology/Principal Findings:**

Here two hexaploid triticales with great morphologic divergence derived from common wheat cultivar M8003 (*Triticum aestivum* L.) × Austrian rye (*Secale cereale* L.) were reported, exhibiting high resistance for powdery mildew and stripe rust and potential for wheat improvement. Sequential fluorescence *in situ* hybridization (FISH) and genomic *in situ* hybridization (GISH) karyotyping revealed that D-genome chromosomes were completely eliminated and the whole A-genome, B-genome and R-genome chromosomes were retained in both lines. Furthermore, plentiful alterations of wheat chromosomes including 5A and 7B were detected in both triticales and additionally altered 5B, 7A chromosome and restructured chromosome 2A was assayed in N9116H and N9116M, respectively, even after selfing for several decades. Besides, meiotic asynchrony was displayed and a variety of storage protein variations were assayed, especially in the HMW/LMW-GS region and secalins region in both triticales.

**Conclusion:**

This study confirms that whole D-genome chromosomes could be preferentially eliminated in the hybrid of common wheat × rye, “genome shock” was accompanying the allopolyploidization of nascent triticales, and great morphologic divergence might result from the genetic variations. Moreover, new hexaploid triticale lines contributing potential resistance resources for wheat improvement were produced.

## Introduction

Polyploidy is a prominent process in plant speciation, and numerous important crop species are polyploid, most importantly allopolyploid [1–3]. As newly synthesized allopolyploids, triticales (× *Triticosecale* sp. *Wittmack*) were derived from Triticeae × rye (*Secale cereale* L.) in a variety of ploidy levels and genome constitutions, such as tetraploid triticale, hexaploid triticale and octoploid triticale [4–7]. Triticales have not only been recognized to have potential as a food cereal and promising in energy supply because of their high biomass and grain yield, but also can serve as significant germplasm resources contributing tolerant genes to both abiotic and biotic stresses in wheat cultivar improvement [4,7–10].

Hexaploid triticales are believed to be more successful than octoploid triticales, on account of their greater meiotic stability and fertility [11–13]. Apart from directly synthesized hexaploid triticales by crossing tetraploid wheat with rye, secondary hexaploid triticales were also developed by crossing an octoploid triticale and/or hexaploid wheat with a hexaploid triticale [14], and many hexaploid derivatives can spontaneously appear in octoploid triticales, which stem from partial elimination of wheat and rye chromosomes [15–19]. In addition, Hao et al. [20] reported that hexaploid triticale with intact A-genome, B-genome and R-genome chromosomes and hexaploid triticales with aberrant chromosomes could be effectively produced via hybridization of synthetic hexaploid wheat with rye, as a result of the elimination of D-genome chromosomes.

Rapid genetic and epigenetic changes have been widely investigated in a series of newly synthesized allopolyploids, such as allotetraploid or allohexaploid cotton, allotetraploid *Arabidopsis*, allotetraploid *Brassica napus*, allotetraploid or allohexaploid wheat and allohexaploid triticale [21–27]. In triticale, it was revealed that whole-chromosome or whole-genome elimination could occur [17,19,20], tandem repeats, regulatory elements and promoter regions were eliminated or expanded [28], both retrotransposon-related and coding sequences could be rearranged [29], AFLP/RFLP-level genomic sequence change was investigated [24,30] and high-molecular-weight glutenin changes stemmed from mitotic illegitimate recombination [31]. However, few researchers have investigated that similar hexaploid triticales could be derived from both common wheat × rye, as well as that “genome shock” was accompanying the derived triticales.

In the present study, we identified the chromosome constitutions of two hexaploid lines derived from common wheat cultivar M8003 × Austrian rye. The two lines were definitely confirmed to be both hexaploid triticales with whole A-genome, B-genome and R-genome chromosomes, while visible phenotypic divergences were present between them. The results of our study indicate that D-genome chromosomes were preferentially eliminated in the hybrid of common wheat × rye, and hexaploid triticales could be generated in common wheat × rye. A similar result was reported earlier [20], but the hexaploid wheat lines used in that work were synthetic wheats, derived from tetraploid wheat x *Aegilops tauschii* accessions. In our study, we began with a hexaploid wheat cultivar suggesting that the results of Hao et al. [20] were not due to any feature of the synthetic wheats.

## Materials and Methods

### Plant materials

Common wheat cultivar M8003, Chinese Spring (CS), Xiaoyan No.6 (*Triticum aestivum* L., 2n = 6× = 42, AABBDD) and Austrian rye (*Secale cereale* L., 2n = 2× = 14, RR) were procured from the College of Agronomy, Northwest A & F University. M8003 was derived from monosomic 5B of CS and Xiaoyan No.6.

**Fig 1:**
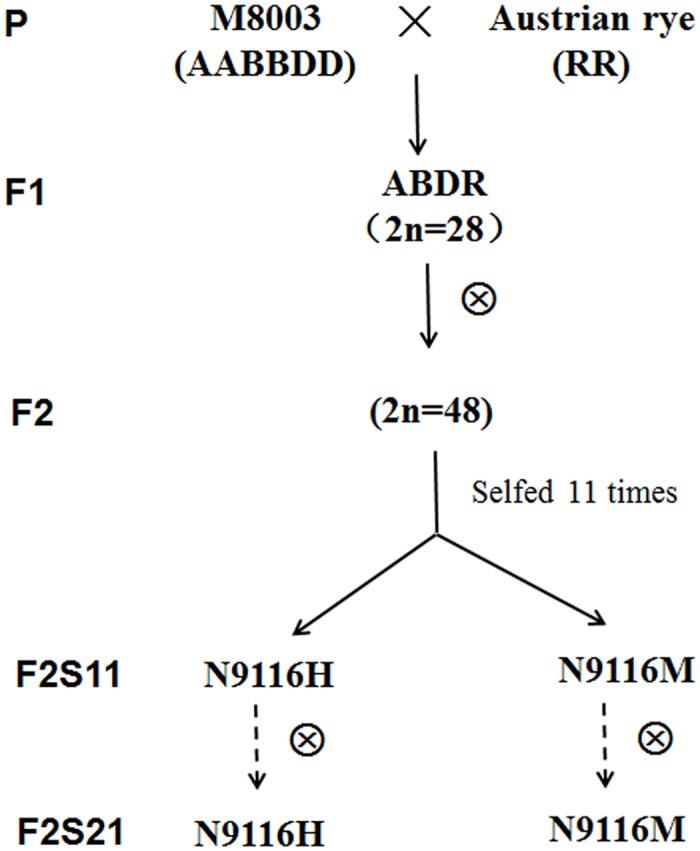
Breeding scheme showing the development of N9116H and N9116M.

The hexaploid triticales were developed according to the breeding scheme shown in Fig. 1. A cross between M8003 and Austrian rye was made in 1991. Although most of the F1 plants were sterile, 6 F2 seeds were fortunately obtained and a F2 seedling carrying 48 chromosomes was recorded to be the parent of N9116H and N9116M in 1992. After 11 times’ selfing, two stable-phenotyped lines were obtained in 2003, namely N9116H and N9116M, respectively. In 2013, N9116H and N9116M have been self-pollinated for 10 years to ensure the stability. All plant materials were maintained by strict selfing in the field or greenhouse of Northwest A & F University.

### Cytological observations

Sequential fluorescence *in situ* hybridization (FISH) and genomic *in situ* hybridization (GISH) were carried out on mitotic chromosome spreads of M8003, Austrian rye, N9116H and N9116M, respectively. Chromosome spreads of materials, probe labeling and *in situ* hybridization were prepared according to the methods previously described by Han et al. [32] and Li et al. [33]. Oligonucleotide probes, Oligo-pTa535 and Oligo-pSc119.2, were 5’ end-labelled with 6-carboxyfluorescein (6-FAM) or 6-carboxytetramethylrhodamine (Tamra), synthesized by Shanghai Invitrogen Biotechnology Co. Ltd. (Shanghai, China), as described by Tang et al. [34]. The genomic DNA of Austrian rye was labeled with Texas Red-5-dUTP (Invitrogen). Photographs were taken with MetaMorph Research Imaging Software (Molecular Devices, Sunnyvale, Calif.) on Olympus BX61 fluorescence microscope, and then processed with Adobe Photoshop CS 3.0.

For meiotic studies, one or two spike(s) were collected from each three different plants to demonstrate the meiotic process, and anthers with pollen mother cells were fixed in Carnoy’s 6:3:1 (ethanol/acetic-acid/chloroform) fixative, and screened using the conventional acetocarmine procedure according to Li et al. [33].

### Morphology and disease resistance evaluation

During the 2013–2014 growing season, field trial evaluations were conducted at the field of Northwest A & F University. Plant height, spike length, kernels per spike and seed setting were recorded from 10 randomly-selected plants at maturity, and average value for each trait was then calculated.

M8003, Austrian rye, N9116H and N9116M were evaluated for adult-plant resistance to stripe rust and powdery mildew at the field of Northwest A & F University in the natural conditions, which were inoculated with races of *Puccinia striiformis* f. sp. *tritici* (CYR31 and CYR32).

### Seed storage protein electrophoresis

The seeds were halved and the portions without embryos were processed to extract their glutenins and gliadins, then sodium dodecyl sulfate polyacrylamide-gel electrophoresis (SDS-PAGE) analysis was conducted as described by Zhao et al. [35], with little modification.

## Results

### Morphologic and cytological observation of hexaploid triticales—N9116H and N9116M

**Fig 2:**
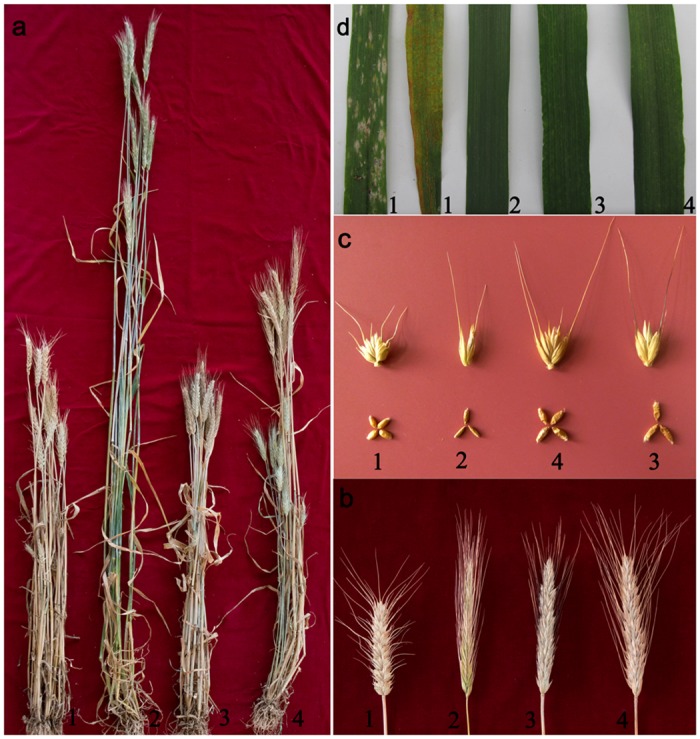
Morphologic traits of M8003, Austrian rye, N9116H and N9116M. a Plant of M8003, Austrian rye, N9116M and N9116H; b Spikes of M8003, Austrian rye, N9116M and N9116H; c Spikelets and kernels of M8003, Austrian rye, N9116H and N9116M; d Resistance of M8003, Austrian rye, N9116H and N9116M for powdery mildew and stripe rust. 1–4 in figures represent M8003, Austrian rye, N9116M and N9116H, respectively.

Two stable hexaploid lines with great phenotypic divergence were obtained in 2003, namely N9116H and N9116M, and the morphology was stably inherited after 11-time sequential selfing. Although N9116H and N9116M displayed typical morphologic traits similar with triticale, adult plant height of N9116M (86.0 cm) was unexpectedly much lower than N9116H (110.7 cm) and the spike length of N9116H (13.9 cm) was a little longer than N9116M (12.2 cm), while awn of N9116M was a little black in comparison with N9116H and their parents’ white awn (Fig. 2 and Table 1). Additionally, both N9116H and N9116M carried more spikelets than M8003, which was similar with Austrian rye. The kernels of N9116H and N9116M were shriveled and longer than those of both parents and both N9116H and N9116M displayed partially sterile. Despite that kernels per spike of N9116H (77.33) were much more than N9116M (53.50), seed-setting of N9116H was worse than N9116M, even as low as 58.67%.

**Table 1:** Morphologic traits of hexaploid triticales N9116H/M and their parents.

Line	Plant height (cm)	Length of leaf (cm)	Length of spike (cm)	No. of spikelet per spike	Kernels per spike	Seed setting (%)
M8003	92.5	19.3	10.0	16.5	48.33	96.94 (96.7–97.2)
Australia rye	121.2	16.7	11.3	31.0	—	—
N9116M	86.0	23.6	12.2	27.1	53.50	71.26 (67.2–74.6)
N9116H	110.7	20.7	13.9	30.6	77.33	58.67 (52.0–64.7)

**Fig 3:**
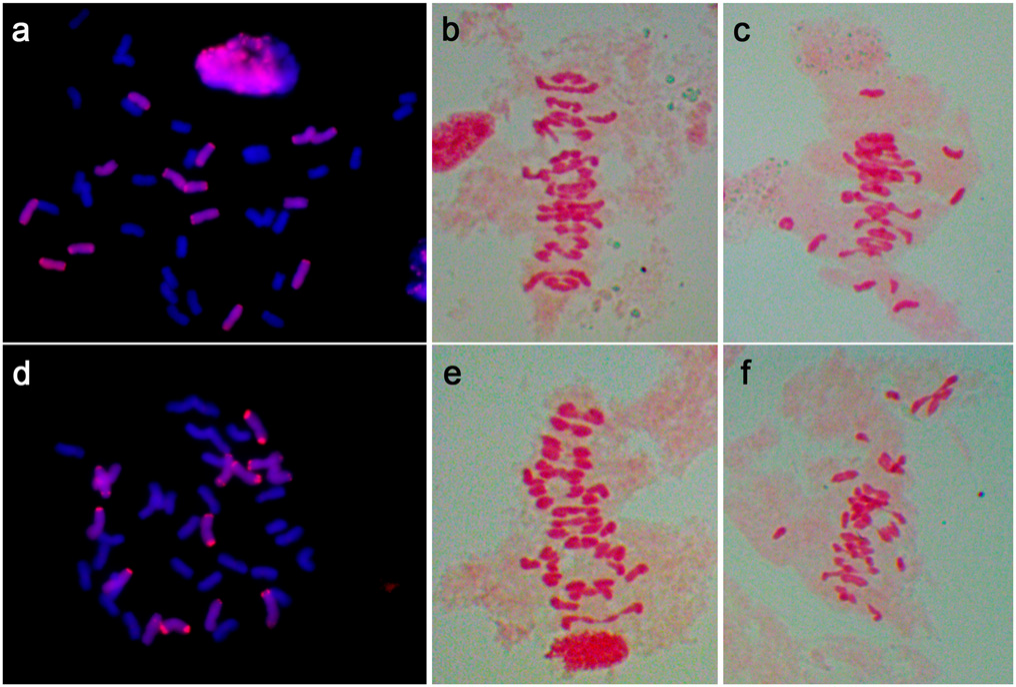
Mitotic and meiotic analysis of N9116H and N9116M. a-c Mitotic and meiotic analysis of N9116H, a 14 rye chromosomes in mitotic cell of N9116H; b,c Meiotic cell of N9116H showing 21 II and 17 II + 8 I, respectively; d-f Mitotic and meiotic analysis of N9116M, d 14 rye chromosomes in mitotic cell of N9116M; e,f Meiotic cell of N9116M showing 21 II and 19 II + 4 I, respectively. a, d 4',6-diamidino-2-phenylindole (DAPI), blue fluorescence; rye genomic DNA, red fluorescence.

Moreover, mitotic and meiotic analyses were carried out to investigate the genome stability of N9116H and N9116M, and somatic chromosome number of both lines were 42, containing 28 wheat chromosomes and 14 rye chromosomes without any rye-wheat translocation chromosomes, demonstrated by GISH analysis with Austrian rye genomic DNA as a probe (Fig. 3a,d).

One or two spike(s) were collected from each three different plants to demonstrate the meiotic process, and it was observed that chromosome configurations of N9116H and N9116M were asynchronous with high percentage of univalents in meiotic metaphase I of pollen mother cells. Specifically, only 45.4% and 71.5% of examined pollen mother cells showed 21 II in N9116H and N9116M, respectively, and up to 8 univalents were observed in N9116H (Fig. 3b,c,e,f and Table 2). However, few mis-divided chromosomes were observed in both lines at the anaphase I of pollen mother cell, regardless of asynchrony in metaphase I. Hence, meiotic asynchrony is generally associated with the nascent hexaploid triticales.

**Table 2:** Numbers and percentages of different chromosome configurations of N9116H and N9116M in metaphase I of first meiotic division.

Line	Chromosome configuration					
	21 II	20 II + 2 I	19 II + 4 I	18 II + 6 I	17 II + 8 I	Total
**N9116H**	188 (0.454)	151 (0.365)	67 (0.162)	7 (0.017)	1 (0.002)	414
**N9116M**	186 (0.715)	65 (0.25)	9 (0.035)	0 (0)	0 (0)	260

Note: The number in parentheses is the portion of the pollen mother cells for each position.

As shown in Fig. 2d, the resistance results showed that M8003 was highly susceptible, whereas Austrian rye, N9116H and N9116M were immune to the tested isolates, indicating that both triticales might inherit the genes for powdery mildew and stripe rust resistance donated by Austrian rye.

### Similar whole-chromosome constitution of N9116H and N9116M karyotyped by sequential FISH and GISH

**Fig 4:**
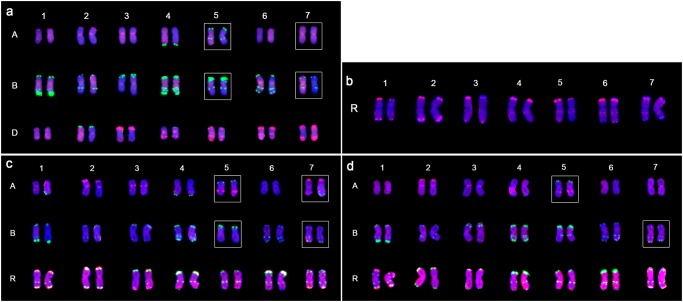
Sequential FISH and GISH karyotypes of M8003 (a), Austrian rye (b), N9116H (c) and N9116M (d). (a, c, d) 4',6-diamidino-2-phenylindole (DAPI), blue fluorescence; rye genomic DNA and Oligo-pTa535, red fluorescence; Oligo-pSc119.2, green fluorescence. (b) Oligo-pSc119.2, red fluorescence. Alterations of wheat chromosomes were indicated in white box.

Inspired by that FISH probes—tandem repeat sequence pTa-535 and pSc119.2 were effective to identify wheat A-, B-, and D-genome chromosomes [34] and pSc119.2 could also discriminate R-genome chromosomes [36], chromosome constitutions of the lines were further demonstrated by sequential FISH and GISH analyses. FISH karyotypes of M8003 and Austrian rye were primarily established by employing Oligo-pTa-535/ pSc119.2 and pSc119.2, respectively (Fig. 4a, b). Then, each 5 plants of N9116H and N9116M were successfully karyotyped by combining Oligo-pTa-535, Oligo-pSc119.2 and rye’s genomic DNA as probes (Fig. 4c, d). Compared to the karyotypes of M8003 and Austrian rye, both triticales displayed similar chromosome constitutions and consisted of whole A-genome, B-genome and R-genome chromosomes instead of any D-genome’s chromosomes. Thus, all the D-genome chromosomes were completely eliminated during the derivation of two hexaploid triticales.

More interestingly, some chromosomes’ FISH signal patterns of N9116H and N9116M differed from their parents’ to some extent, especially the A-genome and B-genome’s chromosomes, which indicate alterations of wheat chromosomes. Specifically, 5AL arm of both triticales displayed Oligo-pSc119.2 signal loss and intercalary Oligo-pTa535 signal gain. 7BL arm of both triticales contained intercalary terminal Oligo-pSc119.2–1 signal, In addition, 7AS of N9116H presented Oligo-pTa535 signal enhancement in the terminal region of short arm, while chromosomes 7A of N9116M were consistent with M8003. 5BL of N9116H instead of N9116M showed Oligo-pSc119.2 signal loss. However, no altered rye chromosome was detected in both triticales.

**Fig 5:**
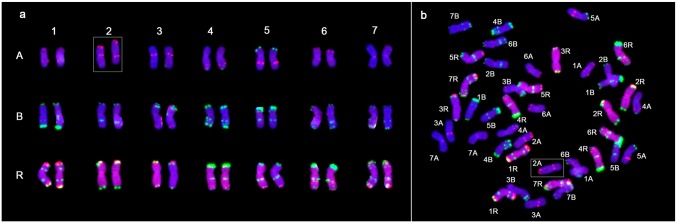
Restructured 2A chromosome detected in N9116M’s plant. 4',6-diamidino-2-phenylindole (DAPI), blue fluorescence; rye genomic DNA and Oligo-pTa535, red fluorescence; Oligo-pSc119.2, green fluorescence. The chromosome in the box indicates restructured 2A chromosome.

Moreover, a restructured chromosome was detected in only a single N9116M’s plant although five plants of each N9116H and N9116M from the same generation were karyotyped. As shown in Fig. 5, the profile of one chromosome 2A was visually different from the other, that is, an intrachromosome aberration containing the extended short arm and shortened long arm was observed although the Oligo-pTa535 signals were maintained a consistent.

Therefore, N9116H and N9116M were demonstrated to be hexaploid triticales carrying whole A-genome, B-genome and R-genome’s chromosomes with complete elimination of D-genome’s chromosomes and plentiful structural alterations of wheat chromosomes were observed instead of rye chomosomes.

### Storage protein variations detected in N9116H and N9116M

Given that the storage proteins of triticale are comprised of high-molecular-weight glutenin subunits (HMW-GS), low-molecular-weight glutenin subunits (LMW-GS), high-molecular-weight (HMW) secalins and α-, β- and γ-gliadins [37–39], the storage proteins of N9116H and N9116M were investigated.

**Fig 6:**
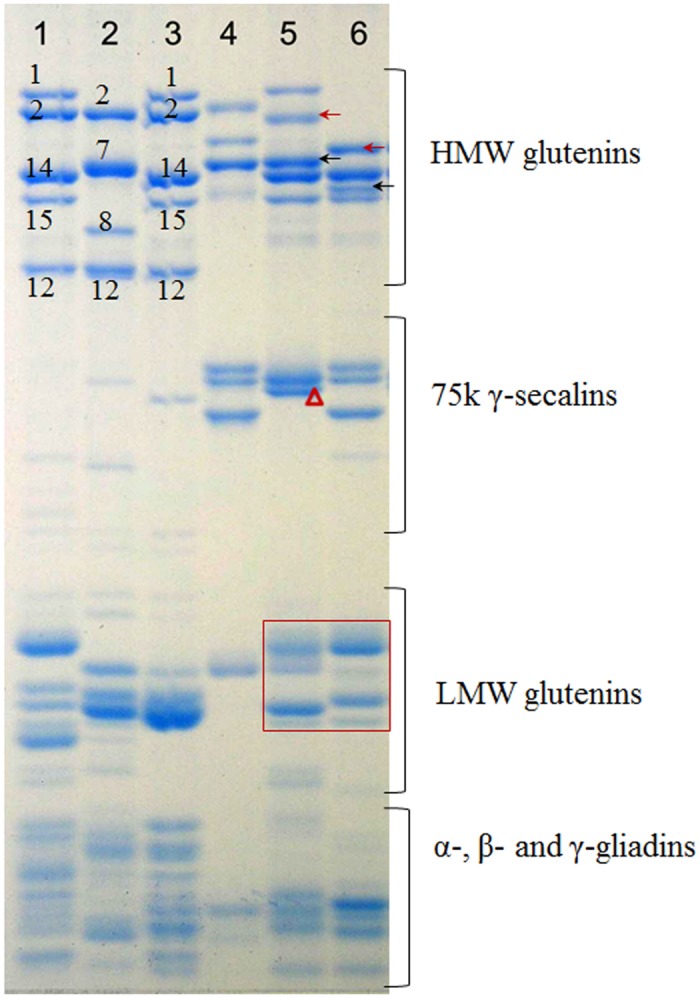
Storage protein variations in hexaploid titicales N9116H and N9116M. 1–6 represent Xiaoyan No.6, Chinese Spring, M8003, Austrian rye, N9116H and N9116M, respectively. Black arrow, position of inherited HMW-GS subunit of Austrian rye; red arrow, novel HMW-GS subunit emerged in N9116H and N9116M; red triangle, novel secalins emerged in N9116H; red box, HMW-GS variations in N9116H and N9116M.

Because of the co-dominance of *Glu-1* alleles, every functional allele encodes a distinguishable HMW-GS and the HMW secalins of rye consisted of x-type and y-type subunits [40]. The composition of M8003’s allelic HMW-GS was determined to be 1Ax1, 1Bx14+1By15, 1Dx2+1Dy12, according to the composition of common wheat cultivar Chinese Spring and Xiaoyan No.6. Interestingly, multiple HMW-GS variations were observed in both triticales. As shown in Fig. 6, 1Bx14+1By15 encoded by 1B chromosome was detectable and 1Dx2+1Dy12 was not present in both triticales owing to the elimination of 1D chromosomes, while 1Ax1 encoded by 1A chromosome was only detected in N9116H rather than both triticales, which indicates the gene silencing of Glu-1Ax allele. In addition, not all the HMW secalins of Austrian rye were simultaneously expressed, however, a single but inconsistent pattern was inherited in N9116H and N9116M, respectively (Fig. 6, indicated by black arrow), and novel but inconsistent patterns were also detected in both triticales (Fig. 6, indicated by red arrow).

In the 75k γ-secalins region, N9116M displayed a similar pattern with Austrian rye whereas a novel pattern was shown in N9116H (Fig. 6, indicated by red triangle). Similarly, polymorphic patterns were also detected between both triticales in the LMW-GS region (Fig. 6, red box). However, no distinct divergence could be distinguished in α-, β- and γ-gliadins region.

Thus, these results confirmed that complicated storage proteins variations were distinguished between N9116H and N9116M and differential expression of glutenin or gliadin could result from gene silencing of the alleles, although similar whole-chromosome constitution was carried.

## Discussion

### Emergence of hexaploid triticale by complete elimination of D-genome chromosomes

This present study demonstrated that hexaploid triticale with whole A-genome, B-genome and R-genome chromosomes could be generated in common wheat × rye. Intriguingly, elimination of the whole D-genome chromosomes has been rarely reported in hybridization of common wheat and rye, apart from similar phenomenon occurring in synthesized hexaploid wheat × rye [20].

Because of the meiotic instability and high aneuploid frequency in primary octoploid triticales [15,41], previous studies showed that hexaploid lines could be spontaneously derived from primary octoploid triticales, with the retention of most of A-, B- and R-genome chromosomes and the elimination of most of the D-genome chromosomes [16,17,19]. Consistently, this study demonstrates that the whole D-genome could be preferentially eliminated in progenies of common wheat × rye. Thus, compared with A, B, and R genomes, the D-genome showed the most lability for chromosome variations in triticales. In synthesized hexaploid wheat, Zhang et al. [42] illustrated that the latest added D subgenome was largely stable in the three constituent subgenomes, while Hao et al. [20] reported that most of the D-genome chromosomes were eliminated when crossing synthesized hexaploid wheat with rye and Tang et al [47] reported wheat chromosomal alterations could be easily induced by rye chromosomes in wheat-rye hybrids, which imply that the stability of the wheat genome could be effectively suppressed by the R genome.

Furthermore, three processes were reported to be associated with chromosome elimination in octoploid triticale, the budding-like chromatin elimination from pollen mother cells [43], unequal chromosome division in somatic cell [44] and centromere loss of chromosome fragments [20]. Our results confirmed that chromosomal variations and asynchronous chromosome-division did accompany the hexaploid triticales even after several decades of selfing. Hence, mitotic and meiotic instability did exist in both nascent hexaploid or octoploid triticales and the reason for this required to be further studied.

### Divergent morphological traits may result from “genome shock” accompanying the allopolyploidization of nascent triticales

Genomic changes could result from interspecific hybridization, known as “genome shock” [45], and chromosome-level perturbation has been manifested in nascent allopolyploidization [42]. In wheat-rye hybrids, high level of genomic changes were presented, such as AFLP/RFLP-level genomic sequence change [14,24,30], the elimination or expansion of tandem repeats, regulatory elements and promoter regions [28] and novel high-molecular-weight glutenins [31]. Previously, extensive restructured chromosomes were observed in newly formed tetraploid wheat [46] and plentiful structural alterations of wheat chromosomes were reported when backcrossing octoploid triticale with common wheat [47]. Similarly, the altered 5A and 7B chromosome were detected in both triticales and additionally altered 5B, 7A chromosome and restructured chromosome 2A was assayed in N9116H and N9116M, respectively, the chromosome 2A of which may carry a translocation of long arm to short arm, which demonstrate the chromosome instability of nascent triticales.

Moreover, a variety of storage protein variations were detected in this study, especially in the HMW/LMW-GS region and secalins region. As observed, not all of the patterns of parents could be inherited, even though the assigned chromosomes of alleles were carried. An illuminating explanation to the phenomenon is that gene silencing or deletion accompanied the storage protein alleles. Besides, novel patterns that distinctly differentiate from the parents also emerged, which indicates that novel gene expressions were activated or coding sequences were rearranged. Although illegitimate recombination is considered to be possible to create novel active alleles or a critical deletion [31], the exact mechanisms still remain to be clarified.

Although these two triticales were derived from the same pedigree and consistent chromosome constitution, great morphological divergences were displayed in both lines. Interestingly, chromosome reorganization, meiotic asynchrony and storage protein variations were also detected in both lines. Hence, great morphological divergences are considered to associate with the genome shock in the nascent triticales.

### The potential of hexaploid triticales derived from common wheat × rye

The present study indicates that hexaploid triticales consisting of whole A-gnome, B-genome and R-genome’s chromosomes can be produced by crossing common wheat and rye. Due to plentiful variations accompanying nascent allopolyploid, these triticales are desirable materials for studying the evolutionary biology of distant hybridization.

As the occurrence and spread of devastating disease could lead to wheat yields losses and grain quality decrease, stripe rust and powdery mildew are economically worldwide threats in wheat growing areas [48,49]. Consequently, it is urgent to search for and transfer novel and effective sources of resistance, while relative species of common wheat are considered potential resources to enhance the resistance during wheat improvement. Here, we showed that both hexaploid triticales were immune to the tested isolates and thereby carried genes for powdery mildew and stripe rust resistance donated by Austrian rye. Both triticales will be valuable bridge resources for resistance genes transfer. Thus, this study not only provides new triticale lines but also contributes to potential resistance resources which can be used for wheat improvement.
